# Crystal structure, Hirshfeld surface analysis and DFT studies of 1,3-bis­[2-meth­oxy-4-(prop-2-en-1-yl)phen­oxy]propane

**DOI:** 10.1107/S2056989020001681

**Published:** 2020-02-14

**Authors:** Abdelmaoujoud Taia, Mohamed Essaber, Tuncer Hökelek, Abdeljalil Aatif, Joel T. Mague, Ali Alsalme, Nabil Al-Zaqri

**Affiliations:** a Laboratory of Molecular Chemistry, Department of Chemistry, Faculty of Sciences Semlalia, University of Cadi Ayyad, PB. 2390, 40001 Marrakech, Morocco; b Department of Physics, Hacettepe University, 06800 Beytepe, Ankara, Turkey; c Department of Chemistry, Tulane University, New Orleans, LA 70118, USA; d Department of Chemistry, College of Science, King Saud University, P.O.Box 2455, Riyadh 11451, Saudi Arabia; e Department of Chemistry, College of Science, King Saud University, PO Box 2455, Riyadh 11451, Saudi Arabia

**Keywords:** crystal structure, all­yl, meth­oxy­phen­oxy, C—H⋯π(ring), Hirshfeld surface

## Abstract

The asymmetric unit of the title compound, C_23_H_28_O_4_, comprises two half-mol­ecules, with the other half of each mol­ecule being completed by the application of twofold rotation symmetry. The two completed mol­ecules both have a V-shaped appearance but differ in their conformations. In the crystal, each independent mol­ecule forms chains extending parallel to the *b* axis with its symmetry-related counterparts through C—H⋯π(ring) inter­actions. Hirshfeld surface analysis of the crystal structure indicates that the most important contributions for the crystal packing are from H⋯H (65.4%), H⋯C/C⋯H (21.8%) and H⋯O/O⋯H (12.3%) inter­actions. Optimized structures using density functional theory (DFT) at the B3LYP/6–311 G(d,p) level are compared with the experimentally determined mol­ecular structures in the solid state. The HOMO–LUMO behaviour was elucidated to determine the energy gap.

## Abstract 2

Two different mol­ecules with point group symmetry 2 are present in the crystal structure of the title compound. Each independent mol­ecule forms chains parallel to the *b* axis with its symmetry-related counterparts through C—H⋯π(ring) inter­actions.

## Chemical context   

Eugenol (4-allyl-2-meth­oxy­phenol) is the main active constituent of clove oil (75–90%) from various plants (Patra & Saxena, 2010 ▸). The 4-allyl-2-meth­oxy­phenol core has several active sites and provides a great responsiveness, making it an excellent precursor in the syntheses of new heterocyclic compounds (Araújo *et al.*, 2010 ▸; Xu *et al.*, 2006 ▸) and for the development of drugs (Sticht & Smith, 1971 ▸). With respect to the biological applications of eugenol 4-allyl-2-meth­oxy­phenol derivatives, it has been shown that these compounds possess potent anti­microbial (Eyambe *et al.*, 2011 ▸), anti­oxidant (Nam & Kim, 2013 ▸; Mahapatra *et al.*, 2009 ▸; Eyambe *et al.*, 2011 ▸), anti­viral (Sun *et al.*, 2016 ▸), anti-inflammatory (Fonsêca *et al.*, 2016 ▸), anti­diabetic and anti-leishmania (de Morais *et al.*, 2014 ▸) properties. The suppression of melanoma growth caused by eugenol was reported by Ghosh *et al.* (2005 ▸), and the ability of eugenol to act as an *in vivo* radio-protective agent was described by Tiku *et al.* (2004 ▸). Derivatives of eugenol have also been reported, see, for example: Sadeghian *et al.* (2008 ▸); Ma *et al.* (2010 ▸).

As a continuation of our research devoted to the study of *o*-alkyl­ation reactions involving eugenol derivatives, we report herein the synthesis, mol­ecular and crystal structures of the title compound, (I). Hirshfeld surface analysis and a density functional theory (DFT) study carried out at the B3LYP/6–311 G(d,p) level for comparison with the experimentally determined mol­ecular structure.
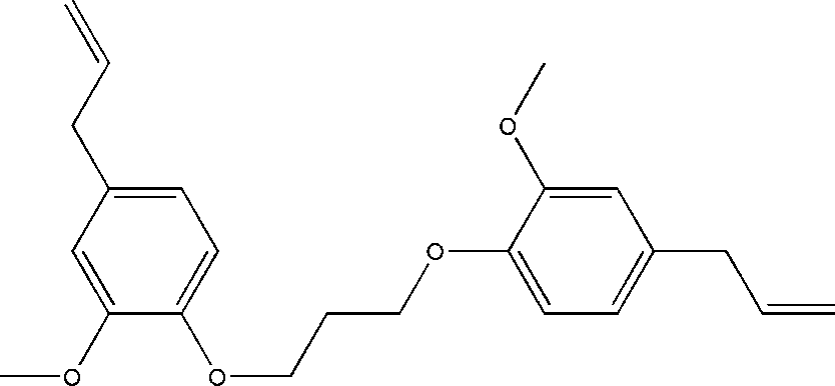


## Structural commentary   

**Figure 1:**
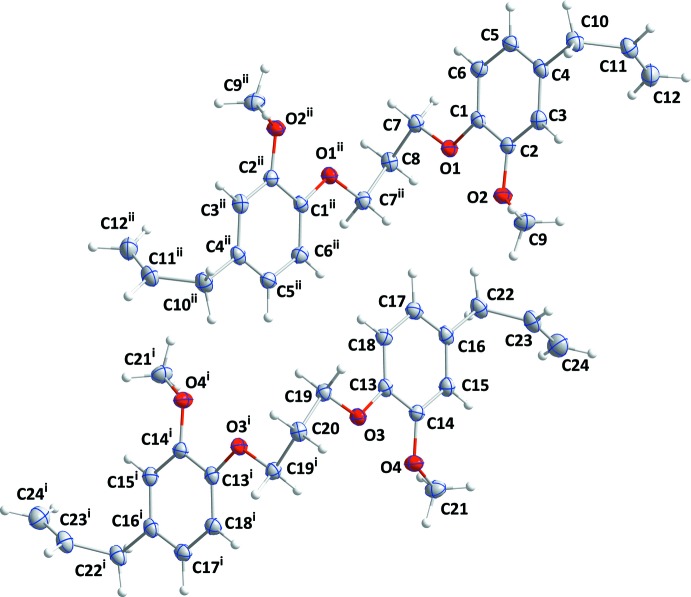
The two independent mol­ecules of (I) with the atom-numbering scheme. Displacement ellipsoids are drawn at the 50% probability level. [Symmetry codes: (i) –*x* + 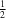, *y*, –*z* + 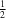; (ii) –*x* + 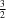, *y*, –*z* + 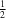.]

**Figure 2:**
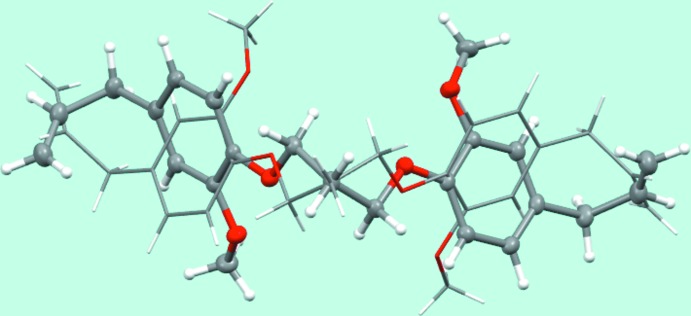
Overlay of the two independent half-mol­ecules, showing their different conformations. Mol­ecule *A* is in light, mol­ecule *B* in dark colours.

The asymmetric unit of (I) comprises of two half-mol­ecules *A* and *B* that are each completed by twofold rotation symmetry, with the rotation axis running through the central C atom (C8 for mol­ecule *A* and C20 for mol­ecule *B*, respectively) of the propane bridge (Fig. 1 ▸). For steric reasons, the exocyclic substituents bound to O1, O2, O3 and O4 are approximately in *trans* positions, with C1—C2—O2—C9, C2—C1—O1—C7, C13—C14—O4—C21 and C14—C13—O3—C19 torsion angles of −167.6 (1), 175.1 (1), 164.6 (1) and −176.7 (1)°, respectively. The two benzene rings in each mol­ecule are nearly perpendicular to each other, with dihedral angles of 86.74 (6)° for *A* (C1–C6) and *A* ^ii^, and of 88.12 (6) for *B* (C13–C18) and *B* ^i^, respectively (for symmetry codes, see Fig. 1 ▸). The two mol­ecules have a similar V-shaped appearance but different conformations (Fig. 2 ▸).

## Supra­molecular features   

**Figure 3:**
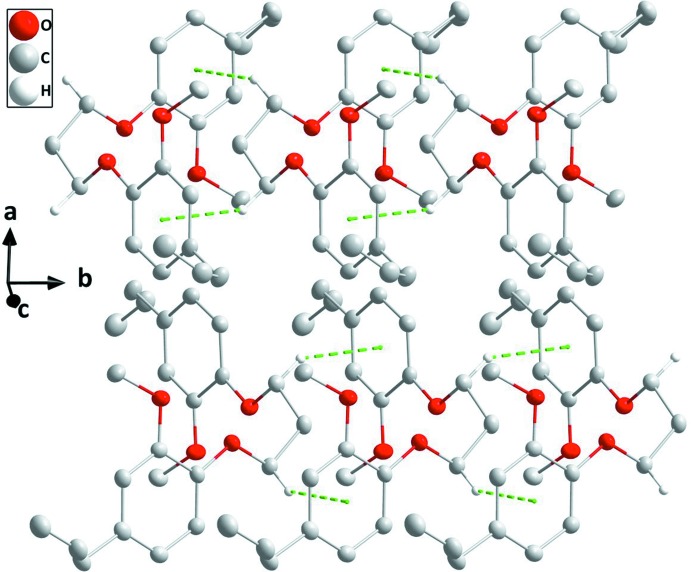
C—H⋯π(ring) inter­actions (green dashed lines) enabling the formation of mol­ecular chains extending along the *b*-axis direction.

In the crystal, chains extending parallel to the *b* axis are formed through C7—H7*A*⋯*Cg*1 (for mol­ecule *A*) and C19—H19*B*⋯*Cg2* (for mol­ecule *B*) inter­actions (Fig. 3 ▸, Table 1 ▸). Between the chains, only van der Waals contacts occur (Figs. 3 ▸ and 4 ▸, Table 2 ▸).

## Hirshfeld surface analysis   

**Figure 5:**
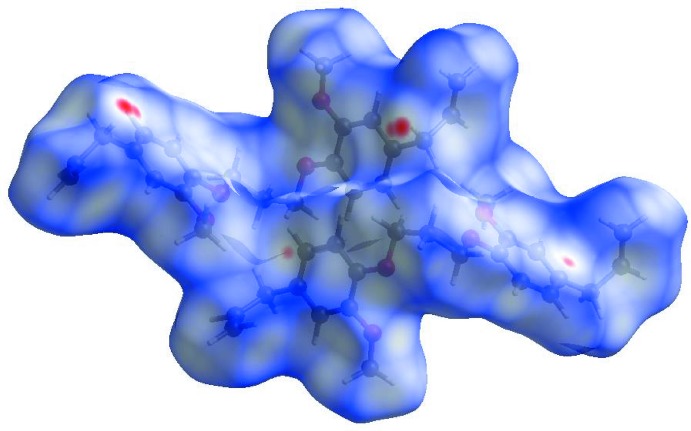
View of the three-dimensional Hirshfeld surface of the title compound plotted over *d*_norm_ in the range −0.1048 to 1.1789 a.u..

**Figure 6:**
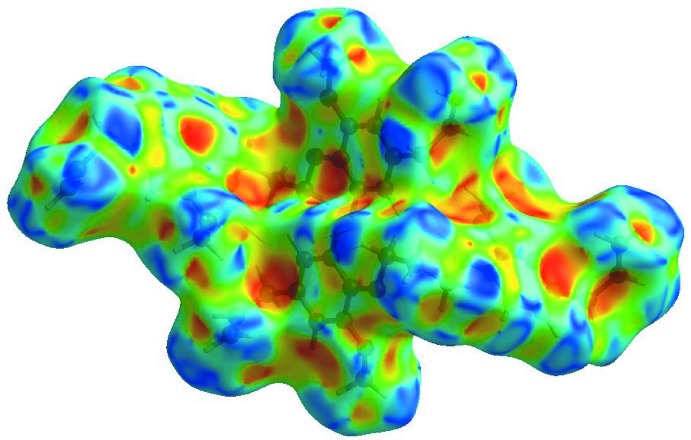
Hirshfeld surface of the title compound plotted over shape-index.

**Figure 7:**
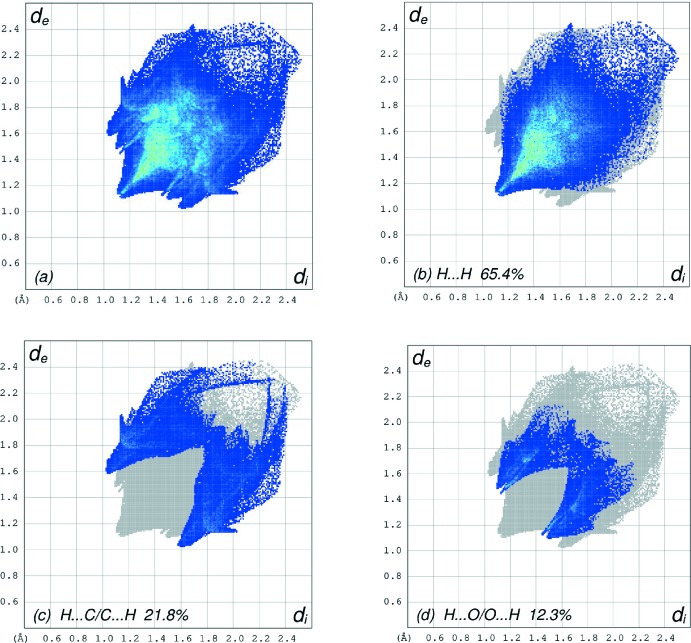
The full two-dimensional fingerprint plots for the title compound, showing (*a*) all inter­actions, and those delineated into (*b*) H⋯H, (*c*) H⋯C/C⋯H and (*d*) H⋯O/O⋯H inter­actions. The *d*_i_ and *d*_e_ values are the closest inter­nal and external distances (in Å) from given points on the Hirshfeld surface contacts.

In order to qu­antify the inter­molecular inter­actions in the crystal of (I), a Hirshfeld surface (HS) analysis (Hirshfeld, 1977 ▸; Spackman & Jayatilaka, 2009 ▸) was carried out using *Crystal Explorer 17.5* (Turner *et al.*, 2017 ▸). In the HS plotted over *d*_norm_ (Fig. 5 ▸), the white surface indicates contacts with distances equal to the sum of van der Waals radii, and the red and blue colours indicate distances shorter (in close contact) or longer (distinct contact) than the van der Waals radii, respectively (Venkatesan *et al.*, 2016 ▸). The bright-red spots appearing near C16 and hydrogen atom H10*B* indicate their roles as the donor and/or acceptor groups in hydrogen-bonding contacts. The shape-index of the HS is a tool to visualize possible π–π stacking inter­actions by the appearance of adjacent red and blue triangles. The absence of such triangles suggests that there are no notable π–π inter­actions in (I) (Fig. 6 ▸). The overall two-dimensional fingerprint plot, Fig. 7 ▸ *a*, and those delineated into H⋯H, H⋯C/C⋯H and H⋯O/O⋯H contacts (McKinnon *et al.*, 2007 ▸) are illustrated in Fig. 7 ▸ *b*–*d*, respectively, together with their relative contributions to the Hirshfeld surface. The most important inter­molecular inter­actions (Table 2 ▸) are H⋯H contacts, contributing 65.4% to the overall crystal packing, which is reflected in Fig. 7 ▸ *b* as widely scattered points of high density due to the large hydrogen content of the mol­ecule with the tip at *d*_e_ = *d*_i_ = 1.11 Å. In the presence of C—H⋯π inter­actions, pairs of characteristic wings with spikes at the tips at *d*_e_ + *d*_i_ = 2.62 Å are seen in the fingerprint plot delineated into H⋯C/C⋯H contacts, Fig. 7 ▸ *c* (21.8% contribution to the HS). Finally, the thin and thick pairs of scattered wings in the fingerprint plot delineated into H⋯O/O⋯H contacts (12.3% contribution), Fig. 7 ▸ *d*, have a symmetrical distribution of points with the edges at *d*_e_ + *d*_i_ = 2.55 and 2.58 Å.

**Figure 8:**
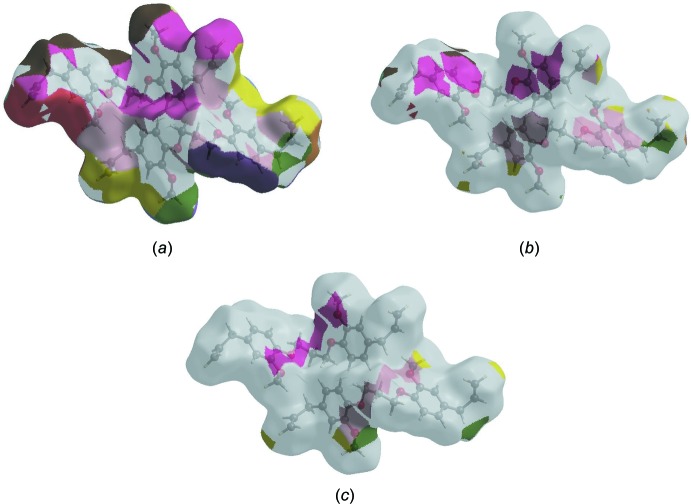
The Hirshfeld surface representations with the function *d*_norm_ plotted onto the surface for (*a*) H⋯H, (*b*) H⋯C/C⋯H and (*c*) H⋯O/O⋯H inter­actions.

Hirshfeld surface representations with the function *d*_norm_ plotted onto the surface are shown for the H⋯H, H⋯C/C⋯H and H⋯O/O⋯H inter­actions in Fig. 8 ▸ *a*–*c*, respectively.

The large number of H⋯H, H⋯C/C⋯H and H⋯O/O⋯H inter­molecular contacts suggest that these weak inter­actions play major roles in the crystal packing (Hathwar *et al.*, 2015 ▸).

## DFT calculations   

The density functional theory (DFT) optimized mol­ecular structures of (I) were computed in the gas phase on the basis of standard B3LYP functionals and 6–311 G(d,p) basis-set calculations (Becke, 1993 ▸) as implemented in *GAUSSIAN 09* (Frisch *et al.*, 2009 ▸). The theoretical and experimental results for mol­ecule *A* are in good agreement (Table 3 ▸).

**Figure 9:**
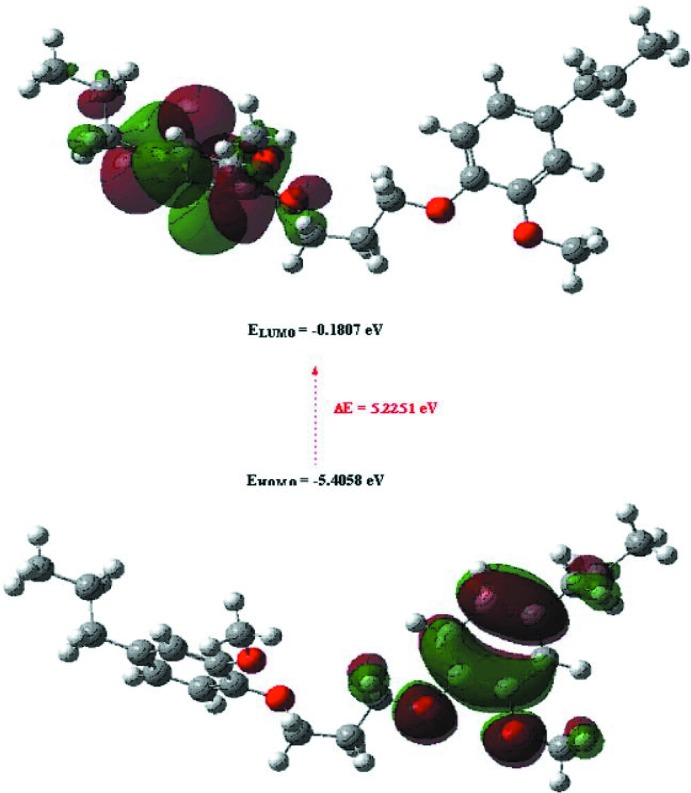
The shapes of HOMO and LUMO orbitals in one of the mol­ecules in (I).

If the energy gap Δ*E* between the highest occupied mol­ecular orbital (HOMO) and the lowest unoccupied mol­ecular orbital (LUMO) is small, the mol­ecule is highly polarizable and has high chemical reactivity. Numerical values of *E*_HOMO_ and *E*_LUMO_, Δ*E* = *E*_LUMO_ - *E*_HOMO_, electronegativity (χ), hardness (η), potential (μ), electrophilicity (ω) and softness (*σ*) for (I) are collated in Table 4 ▸. The significance of η and *σ* is to evaluate both the reactivity and stability. The shapes of the HOMO and the LUMO of mol­ecule *A*, together with their energy levels are shown in Fig. 9 ▸.

## Synthesis and crystallization   

1,3-Di­bromo­propane (0.2 ml, 1.61 mmol) was added to a solution of eugenol (0.5 ml, 3.23 mmol), tetra­butyl­ammonium chloride (50 mg) and sodium hydroxide solution (5%) in benzene as solvent (20 ml). The mixture was stirred at 293 K for 6 h, and then was extracted three times with di­chloro­methane (15 ml). The residue was purified by column chromatography on silica gel using a mixture of hexa­ne/ethyl acetate (*v*/*v* = 97/3) as eluent. Colourless crystals were isolated when the solvent was allowed to evaporate (yield: 86%).

## Refinement   

Details including crystal data, data collection and refinement are summarized in Table 5 ▸. Hydrogen atoms were located in a difference-Fourier map and were refined freely.

## Supplementary Material

Crystal structure: contains datablock(s) I, global. DOI: 10.1107/S2056989020001681/wm5538sup1.cif

Structure factors: contains datablock(s) I. DOI: 10.1107/S2056989020001681/wm5538Isup2.hkl

Supporting information file. DOI: 10.1107/S2056989020001681/wm5538Isup3.cdx

Supporting information file. DOI: 10.1107/S2056989020001681/wm5538Isup4.cml

CCDC reference: 1982222

Additional supporting information:  crystallographic information; 3D view; checkCIF report

## Figures and Tables

**Figure 4:**
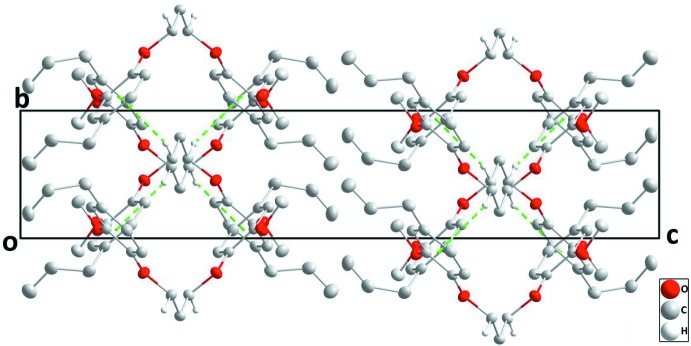
A partial packing diagram viewed along the *a* axis with inter­molecular inter­actions depicted as in Fig. 3 ▸.

**Table 1:** Hydrogen-bond geometry (Å, °) *Cg*1 and *Cg*2 are the centroids of benzene rings *A* (C1–C6) and *B* (C13–C18), respectively.

*D*—H⋯*A*	*D*—H	H⋯*A*	*D*⋯*A*	*D*—H⋯*A*
C7—H7*A*⋯*Cg*1^i^	1.003 (16)	2.759 (15)	3.6170 (15)	144.1 (12)
C19—H19*B*⋯*Cg*2^v^	0.992 (16)	2.739 (15)	3.5816 (15)	143.0 (11)

Symmetry codes: (i) 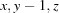; (v) 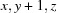.

**Table 2:** Selected interatomic distances (Å)

O1⋯O2	2.5827 (13)	C14⋯H20*B* ^i^	2.918 (15)
O2⋯O1	2.5827 (13)	C15⋯H21*A*	2.768 (17)
O3⋯O4	2.5885 (13)	C15⋯H21*C*	2.784 (17)
O4⋯O3	2.5885 (13)	C17⋯H22*A* ^v^	2.727 (19)
O1⋯H9*C* ^i^	2.736 (18)	C18⋯H22*A* ^v^	2.764 (18)
O1⋯H7*B* ^ii^	2.618 (16)	C18⋯H19*A*	2.759 (15)
O2⋯H12*A* ^iii^	2.831 (18)	C18⋯H19*B*	2.739 (15)
O2⋯H22*B*	2.647 (17)	C19⋯H18	2.514 (16)
O2⋯H8*B* ^iv^	2.676 (15)	C21⋯H15	2.501 (16)
O3⋯H21*A* ^v^	2.739 (15)	C23⋯H15	2.862 (16)
O3⋯H19*A* ^vi^	2.604 (16)	H3⋯H9*C*	2.33 (2)
O4⋯H24*B* ^vii^	2.89 (2)	H3⋯H9*A*	2.29 (2)
O4⋯H20*B* ^i^	2.732 (15)	H5⋯H10*A*	2.37 (2)
C2⋯C7^v^	3.533 (2)	H6⋯H7*A*	2.24 (2)
C3⋯C12	3.282 (2)	H6⋯H7*B*	2.37 (2)
C6⋯C10^i^	3.582 (2)	H6⋯H18^viii^	2.50 (2)
C14⋯C19^i^	3.555 (2)	H9*A*⋯H11^ix^	2.40 (2)
C18⋯C22^v^	3.564 (2)	H9*A*⋯H12*A* ^iii^	2.56 (3)
C2⋯H8*B* ^iv^	2.914 (16)	H9*B*⋯H22*A* ^v^	2.52 (2)
C2⋯H7*A* ^v^	2.971 (15)	H9*B*⋯H23	2.41 (2)
C3⋯H9*C*	2.739 (18)	H9*C*⋯H22*B* ^v^	2.54 (2)
C3⋯H12*B*	2.783 (19)	H10*A*⋯H21*A* ^x^	2.58 (2)
C3⋯H9*A*	2.806 (17)	H12*B*⋯H12*B* ^iii^	2.55 (3)
C4⋯H12*B*	2.728 (18)	H15⋯H21*A*	2.40 (2)
C5⋯H10*B* ^i^	2.775 (19)	H15⋯H21*C*	2.26 (2)
C6⋯H7*A*	2.719 (15)	H17⋯H22*B*	2.36 (2)
C6⋯H7*B*	2.786 (15)	H18⋯H19*A*	2.31 (2)
C6⋯H10*B* ^i^	2.837 (18)	H18⋯H19*B*	2.26 (2)
C7⋯H6	2.529 (16)	H21*C*⋯H23^vii^	2.41 (2)
C9⋯H3	2.492 (16)	H22*A*⋯H24*A*	2.39 (3)

Symmetry codes: (i) 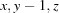; (ii) 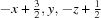; (iii) 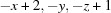; (iv) 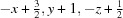; (v) 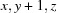; (vi) 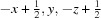; (vii) 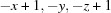; (viii) 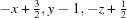; (ix) 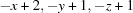; (x) 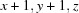.

**Table 3:** Comparison of selected bond lengths and angles (Å, °) in the experinentally determined and computed mol­ecular structures

Bonds/angles	X-ray (this study)	B3LYP/6–311G(d,p)
O1—C1	1.3704 (15)	1.38958
O1—C7	1.4342 (15)	1.46082
O2—C2	1.3679 (15)	1.39236
O2—C9	1.4280 (16)	1.44976
O3—C13	1.3658 (15)	1.39978
O3—C19	1.4359 (15)	1.47837
O4—C14	1.3697 (15)	1.39894
O4—C21	1.4289 (16)	1.45321
		
C1—O1—C7	116.96 (10)	118.14221
C2—O2—C9	116.34 (10)	117.63310
C13—O3—C19	116.88 (10)	117.32223
C14—O4—C21	116.03 (10)	117.85841
O1—C1—C6	125.38 (11)	124.87388
O1—C1—C2	115.50 (11)	116.13060
C6—C1—C2	119.12 (11)	118.99394
O2—C2—C3	124.79 (12)	124.29736
O2—C2—C1	115.21 (11)	115.78966

**Table 4:** Calculated energies and other calculated data for (I)

Total Energy, *TE* (eV)	−32558
E_HOMO_ (eV)	−5.4058
E_LUMO_ (eV)	−0.1807
Gap, *ΔE* (eV)	5.2251
Dipole moment, *μ* (Debye)	2.8076
Ionization potential, *I* (eV)	5.4058
Electron affinity, *A*	0.1807
Electronegativity, *χ*	2.7932
Hardness, *η*	2.6126
Electrophilicity index, *ω*	1.4932
Softness, *σ*	0.3828
Fraction of electrons transferred, *ΔN*	0.8051

**Table 5:** Experimental details

Crystal data	
Chemical formula	C_23_H_28_O_4_
*M*_r_	368.45
Crystal system, space group	Monoclinic, *P*2/*n*
Temperature (K)	150
*a*, *b*, *c* (Å)	15.4741 (5), 5.0224 (2), 25.5180 (9)
β (°)	99.858 (2)
*V* (Å^3^)	1953.90 (12)
*Z*	4
Radiation type	Cu *K*α
μ (mm^−1^)	0.68
Crystal size (mm)	0.37 × 0.27 × 0.08

Data collection	
Diffractometer	Bruker D8 VENTURE PHOTON 100 CMOS
Absorption correction	Multi-scan (*SADABS*; Krause *et al.*, 2015 ▸)
*T*_min_, *T*_max_	0.79, 0.95
No. of measured, independent and observed [*I* > 2σ(*I*)] reflections	13935, 3756, 3049
*R*_int_	0.033
(sin θ/λ)_max_ (Å^−1^)	0.618

Refinement	
*R*[*F* ^2^ > 2σ(*F* ^2^)], *wR*(*F* ^2^), *S*	0.041, 0.109, 1.05
No. of reflections	3756
No. of parameters	358
H-atom treatment	All H-atom parameters refined
Δρ_max_, Δρ_min_ (e Å^−3^)	0.19, −0.23

Computer programs: *APEX3* and *SAINT* (Bruker, 2016 ▸), *SHELXT* (Sheldrick, 2015*a* ▸), *SHELXL2018* (Sheldrick, 2015*b* ▸), *DIAMOND* (Brandenburg & Putz, 2012 ▸) and *publCIF* (Westrip, 2010 ▸).

## supplementary crystallographic information

### Crystal data

**Table:**

C_23_H_28_O_4_	*F*(000) = 792
*M**_r_* = 368.45	*D*_x_ = 1.253 Mg m^−^^3^
Monoclinic, *P*2/*n*	Cu *K*α radiation, λ = 1.54178 Å
*a* = 15.4741 (5) Å	Cell parameters from 9361 reflections
*b* = 5.0224 (2) Å	θ = 3.5–72.4°
*c* = 25.5180 (9) Å	µ = 0.68 mm^−^^1^
β = 99.858 (2)°	*T* = 150 K
*V* = 1953.90 (12) Å^3^	Plate, colourless
*Z* = 4	0.37 × 0.27 × 0.08 mm

### Data collection

**Table:**

Bruker D8 VENTURE PHOTON 100 CMOS diffractometer	3756 independent reflections
Radiation source: INCOATEC IµS micro–focus source	3049 reflections with *I* > 2σ(*I*)
Mirror monochromator	*R*_int_ = 0.033
Detector resolution: 10.4167 pixels mm^-1^	θ_max_ = 72.4°, θ_min_ = 3.1°
ω scans	*h* = −19→18
Absorption correction: multi-scan (*SADABS*; Krause *et al.*, 2015)	*k* = −5→6
*T*_min_ = 0.79, *T*_max_ = 0.95	*l* = −31→29
13935 measured reflections	

### Refinement

**Table:**

Refinement on *F*^2^	Secondary atom site location: difference Fourier map
Least-squares matrix: full	Hydrogen site location: difference Fourier map
*R*[*F*^2^ > 2σ(*F*^2^)] = 0.041	All H-atom parameters refined
*wR*(*F*^2^) = 0.109	*w* = 1/[σ^2^(*F*_o_^2^) + (0.0537*P*)^2^ + 0.5337*P*] where *P* = (*F*_o_^2^ + 2*F*_c_^2^)/3
*S* = 1.05	(Δ/σ)_max_ = 0.001
3756 reflections	Δρ_max_ = 0.19 e Å^−^^3^
358 parameters	Δρ_min_ = −0.23 e Å^−^^3^
0 restraints	Extinction correction: *SHELXL2018* (Sheldrick, 2015*b*), Fc^*^=kFc[1+0.001xFc^2^λ^3^/sin(2θ)]^-1/4^
Primary atom site location: dual	Extinction coefficient: 0.0071 (4)

### Special details

**Table:**

Geometry. All esds (except the esd in the dihedral angle between two l.s. planes) are estimated using the full covariance matrix. The cell esds are taken into account individually in the estimation of esds in distances, angles and torsion angles; correlations between esds in cell parameters are only used when they are defined by crystal symmetry. An approximate (isotropic) treatment of cell esds is used for estimating esds involving l.s. planes.
Refinement. Refinement of F^2^ against ALL reflections. The weighted R-factor wR and goodness of fit S are based on F^2^, conventional R-factors R are based on F, with F set to zero for negative F^2^. The threshold expression of F^2^ > 2sigma(F^2^) is used only for calculating R-factors(gt) etc. and is not relevant to the choice of reflections for refinement. R-factors based on F^2^ are statistically about twice as large as those based on F, and R- factors based on ALL data will be even larger.

### Fractional atomic coordinates and isotropic or equivalent isotropic displacement parameters (Å^2^)

**Table:**

	*x*	*y*	*z*	*U*_iso_*/*U*_eq_	
O1	0.82230 (6)	−0.27068 (18)	0.30688 (3)	0.0222 (2)	
O2	0.79368 (6)	0.06905 (18)	0.37794 (4)	0.0249 (2)	
O3	0.32386 (6)	0.45366 (18)	0.30591 (3)	0.0223 (2)	
O4	0.29863 (6)	0.12078 (19)	0.37925 (4)	0.0258 (2)	
C1	0.89151 (8)	−0.1081 (2)	0.32631 (5)	0.0194 (3)	
C2	0.87636 (8)	0.0744 (2)	0.36573 (5)	0.0203 (3)	
C3	0.94260 (9)	0.2442 (3)	0.38859 (5)	0.0229 (3)	
H3	0.9308 (10)	0.372 (3)	0.4158 (6)	0.026 (4)*	
C4	1.02517 (8)	0.2399 (3)	0.37294 (5)	0.0227 (3)	
C5	1.03861 (8)	0.0650 (3)	0.33324 (5)	0.0234 (3)	
H5	1.0965 (11)	0.061 (3)	0.3204 (6)	0.028 (4)*	
C6	0.97202 (8)	−0.1084 (3)	0.30986 (5)	0.0226 (3)	
H6	0.9834 (10)	−0.232 (3)	0.2825 (6)	0.028 (4)*	
C7	0.83297 (8)	−0.4437 (3)	0.26374 (5)	0.0219 (3)	
H7A	0.8849 (10)	−0.562 (3)	0.2749 (6)	0.023 (4)*	
H7B	0.8441 (10)	−0.334 (3)	0.2325 (6)	0.025 (4)*	
C8	0.750000	−0.6078 (4)	0.250000	0.0224 (4)	
H8B	0.7581 (11)	−0.722 (3)	0.2188 (6)	0.033 (4)*	
C9	0.77076 (10)	0.2853 (3)	0.40922 (6)	0.0289 (3)	
H9A	0.8027 (11)	0.277 (3)	0.4452 (7)	0.034 (4)*	
H9B	0.7090 (11)	0.260 (3)	0.4097 (6)	0.028 (4)*	
H9C	0.7809 (11)	0.458 (4)	0.3922 (7)	0.036 (4)*	
C10	1.09695 (9)	0.4256 (3)	0.39847 (6)	0.0280 (3)	
H10A	1.1474 (11)	0.420 (3)	0.3779 (6)	0.035 (4)*	
H10B	1.0731 (12)	0.613 (4)	0.3959 (7)	0.040 (5)*	
C11	1.13272 (9)	0.3754 (3)	0.45635 (6)	0.0298 (3)	
H11	1.1823 (13)	0.494 (4)	0.4714 (7)	0.052 (5)*	
C12	1.10801 (11)	0.1937 (3)	0.48750 (6)	0.0355 (4)	
H12A	1.1352 (12)	0.181 (4)	0.5248 (7)	0.044 (5)*	
H12B	1.0621 (13)	0.067 (4)	0.4757 (7)	0.047 (5)*	
C13	0.39264 (8)	0.2882 (2)	0.32379 (5)	0.0198 (3)	
C14	0.37979 (8)	0.1110 (2)	0.36473 (5)	0.0204 (3)	
C15	0.44651 (9)	−0.0598 (3)	0.38657 (5)	0.0230 (3)	
H15	0.4371 (11)	−0.180 (3)	0.4150 (6)	0.032 (4)*	
C16	0.52658 (8)	−0.0646 (3)	0.36808 (5)	0.0229 (3)	
C17	0.53771 (8)	0.1037 (3)	0.32686 (5)	0.0235 (3)	
H17	0.5958 (10)	0.104 (3)	0.3123 (6)	0.026 (4)*	
C18	0.47108 (8)	0.2800 (3)	0.30487 (5)	0.0224 (3)	
H18	0.4801 (10)	0.400 (3)	0.2759 (6)	0.023 (4)*	
C19	0.33292 (8)	0.6261 (3)	0.26230 (5)	0.0213 (3)	
H19A	0.3426 (10)	0.514 (3)	0.2309 (6)	0.023 (4)*	
H19B	0.3849 (10)	0.743 (3)	0.2722 (6)	0.026 (4)*	
C20	0.250000	0.7913 (4)	0.250000	0.0223 (4)	
H20B	0.2441 (11)	0.908 (3)	0.2806 (6)	0.031 (4)*	
C21	0.27648 (10)	−0.0984 (3)	0.41011 (6)	0.0289 (3)	
H21A	0.2830 (11)	−0.273 (3)	0.3913 (6)	0.034 (4)*	
H21B	0.2159 (12)	−0.073 (3)	0.4127 (6)	0.036 (4)*	
H21C	0.3109 (11)	−0.096 (3)	0.4452 (7)	0.033 (4)*	
C22	0.59762 (9)	−0.2578 (3)	0.39229 (6)	0.0275 (3)	
H22A	0.5774 (11)	−0.442 (4)	0.3845 (7)	0.037 (4)*	
H22B	0.6510 (11)	−0.225 (3)	0.3753 (6)	0.034 (4)*	
C23	0.62171 (9)	−0.2341 (3)	0.45158 (6)	0.0300 (3)	
H23	0.6524 (13)	−0.064 (4)	0.4645 (7)	0.050 (5)*	
C24	0.60596 (11)	−0.4141 (3)	0.48616 (7)	0.0386 (4)	
H24A	0.5776 (13)	−0.581 (4)	0.4740 (8)	0.051 (5)*	
H24B	0.6230 (13)	−0.389 (4)	0.5240 (8)	0.050 (5)*	

### Atomic displacement parameters (Å^2^)

**Table:**

	*U*^11^	*U*^22^	*U*^33^	*U*^12^	*U*^13^	*U*^23^
O1	0.0208 (4)	0.0227 (5)	0.0230 (5)	−0.0028 (4)	0.0034 (3)	−0.0050 (4)
O2	0.0209 (5)	0.0269 (5)	0.0276 (5)	0.0002 (4)	0.0063 (4)	−0.0056 (4)
O3	0.0212 (4)	0.0223 (5)	0.0232 (5)	0.0035 (4)	0.0032 (3)	0.0049 (4)
O4	0.0225 (5)	0.0274 (5)	0.0289 (5)	0.0031 (4)	0.0084 (4)	0.0061 (4)
C1	0.0191 (6)	0.0183 (6)	0.0197 (6)	−0.0008 (5)	0.0002 (5)	0.0022 (5)
C2	0.0200 (6)	0.0215 (6)	0.0193 (6)	0.0012 (5)	0.0031 (5)	0.0022 (5)
C3	0.0256 (7)	0.0208 (6)	0.0213 (6)	0.0009 (5)	0.0010 (5)	−0.0006 (5)
C4	0.0221 (6)	0.0215 (6)	0.0224 (6)	−0.0019 (5)	−0.0015 (5)	0.0046 (5)
C5	0.0196 (6)	0.0265 (7)	0.0240 (6)	−0.0004 (5)	0.0033 (5)	0.0045 (6)
C6	0.0238 (6)	0.0224 (6)	0.0216 (6)	0.0016 (5)	0.0041 (5)	0.0002 (5)
C7	0.0238 (7)	0.0203 (6)	0.0209 (6)	0.0012 (5)	0.0023 (5)	−0.0026 (5)
C8	0.0247 (9)	0.0188 (8)	0.0223 (9)	0.000	0.0001 (7)	0.000
C9	0.0293 (7)	0.0278 (7)	0.0314 (8)	0.0045 (6)	0.0100 (6)	−0.0045 (6)
C10	0.0265 (7)	0.0263 (7)	0.0293 (7)	−0.0065 (6)	−0.0006 (6)	0.0028 (6)
C11	0.0276 (7)	0.0308 (7)	0.0285 (7)	−0.0026 (6)	−0.0015 (5)	−0.0040 (6)
C12	0.0367 (8)	0.0419 (9)	0.0258 (8)	−0.0008 (7)	−0.0007 (6)	0.0021 (7)
C13	0.0207 (6)	0.0174 (6)	0.0199 (6)	0.0013 (5)	0.0000 (5)	−0.0022 (5)
C14	0.0197 (6)	0.0210 (6)	0.0203 (6)	−0.0007 (5)	0.0033 (5)	−0.0027 (5)
C15	0.0248 (6)	0.0221 (6)	0.0212 (6)	0.0019 (5)	0.0013 (5)	0.0013 (5)
C16	0.0227 (6)	0.0219 (6)	0.0222 (6)	0.0013 (5)	−0.0015 (5)	−0.0052 (5)
C17	0.0209 (6)	0.0240 (6)	0.0254 (7)	−0.0008 (5)	0.0032 (5)	−0.0048 (5)
C18	0.0227 (6)	0.0217 (6)	0.0225 (6)	−0.0020 (5)	0.0033 (5)	−0.0004 (5)
C19	0.0226 (6)	0.0204 (6)	0.0206 (6)	−0.0004 (5)	0.0026 (5)	0.0027 (5)
C20	0.0249 (9)	0.0181 (8)	0.0224 (9)	0.000	0.0001 (7)	0.000
C21	0.0297 (7)	0.0280 (7)	0.0308 (8)	−0.0009 (6)	0.0104 (6)	0.0053 (6)
C22	0.0253 (7)	0.0252 (7)	0.0295 (7)	0.0055 (6)	−0.0019 (5)	−0.0036 (6)
C23	0.0282 (7)	0.0280 (7)	0.0311 (7)	0.0040 (6)	−0.0029 (6)	0.0007 (6)
C24	0.0411 (9)	0.0364 (9)	0.0381 (9)	0.0078 (7)	0.0059 (7)	0.0063 (7)

### Geometric parameters (Å, º)

**Table:**

O1—C1	1.3704 (15)	C11—C12	1.310 (2)
O1—C7	1.4342 (15)	C11—H11	0.99 (2)
O2—C2	1.3679 (15)	C12—H12A	0.975 (19)
O2—C9	1.4280 (16)	C12—H12B	0.96 (2)
O3—C13	1.3658 (15)	C13—C18	1.3816 (18)
O3—C19	1.4359 (15)	C13—C14	1.4121 (17)
O4—C14	1.3697 (15)	C14—C15	1.3840 (18)
O4—C21	1.4289 (16)	C15—C16	1.3995 (18)
C1—C6	1.3812 (18)	C15—H15	0.974 (17)
C1—C2	1.4098 (17)	C16—C17	1.3828 (19)
C2—C3	1.3831 (19)	C16—C22	1.5162 (18)
C3—C4	1.4025 (18)	C17—C18	1.4015 (19)
C3—H3	0.984 (16)	C17—H17	1.030 (15)
C4—C5	1.3833 (19)	C18—H18	0.984 (16)
C4—C10	1.5099 (18)	C19—C20	1.5148 (16)
C5—C6	1.4021 (19)	C19—H19A	1.012 (15)
C5—H5	1.005 (16)	C19—H19B	0.992 (16)
C6—H6	0.972 (16)	C20—H20B	0.993 (16)
C7—C8	1.5154 (16)	C20—H20B^ii^	0.993 (16)
C7—H7A	1.003 (16)	C21—H21A	1.013 (17)
C7—H7B	1.007 (15)	C21—H21B	0.960 (18)
C8—H8B	1.007 (16)	C21—H21C	0.961 (17)
C8—H8B^i^	1.007 (16)	C22—C23	1.499 (2)
C9—H9A	0.967 (17)	C22—H22A	0.984 (18)
C9—H9B	0.966 (17)	C22—H22B	1.010 (17)
C9—H9C	0.993 (18)	C23—C24	1.315 (2)
C10—C11	1.507 (2)	C23—H23	1.00 (2)
C10—H10A	1.014 (17)	C24—H24A	0.97 (2)
C10—H10B	1.008 (18)	C24—H24B	0.97 (2)
			
O1···O2	2.5827 (13)	C14···H20B^iii^	2.918 (15)
O2···O1	2.5827 (13)	C15···H21A	2.768 (17)
O3···O4	2.5885 (13)	C15···H21C	2.784 (17)
O4···O3	2.5885 (13)	C17···H22A^vi^	2.727 (19)
O1···H9C^iii^	2.736 (18)	C18···H22A^vi^	2.764 (18)
O1···H7B^i^	2.618 (16)	C18···H19A	2.759 (15)
O2···H12A^iv^	2.831 (18)	C18···H19B	2.739 (15)
O2···H22B	2.647 (17)	C19···H18	2.514 (16)
O2···H8B^v^	2.676 (15)	C21···H15	2.501 (16)
O3···H21A^vi^	2.739 (15)	C23···H15	2.862 (16)
O3···H19A^ii^	2.604 (16)	H3···H9C	2.33 (2)
O4···H24B^vii^	2.89 (2)	H3···H9A	2.29 (2)
O4···H20B^iii^	2.732 (15)	H5···H10A	2.37 (2)
C2···C7^vi^	3.533 (2)	H6···H7A	2.24 (2)
C3···C12	3.282 (2)	H6···H7B	2.37 (2)
C6···C10^iii^	3.582 (2)	H6···H18^viii^	2.50 (2)
C14···C19^iii^	3.555 (2)	H9A···H11^ix^	2.40 (2)
C18···C22^vi^	3.564 (2)	H9A···H12A^iv^	2.56 (3)
C2···H8B^v^	2.914 (16)	H9B···H22A^vi^	2.52 (2)
C2···H7A^vi^	2.971 (15)	H9B···H23	2.41 (2)
C3···H9C	2.739 (18)	H9C···H22B^vi^	2.54 (2)
C3···H12B	2.783 (19)	H10A···H21A^x^	2.58 (2)
C3···H9A	2.806 (17)	H12B···H12B^iv^	2.55 (3)
C4···H12B	2.728 (18)	H15···H21A	2.40 (2)
C5···H10B^iii^	2.775 (19)	H15···H21C	2.26 (2)
C6···H7A	2.719 (15)	H17···H22B	2.36 (2)
C6···H7B	2.786 (15)	H18···H19A	2.31 (2)
C6···H10B^iii^	2.837 (18)	H18···H19B	2.26 (2)
C7···H6	2.529 (16)	H21C···H23^vii^	2.41 (2)
C9···H3	2.492 (16)	H22A···H24A	2.39 (3)
			
C1—O1—C7	116.96 (10)	C11—C12—H12B	123.1 (11)
C2—O2—C9	116.34 (10)	H12A—C12—H12B	115.9 (15)
C13—O3—C19	116.88 (10)	O3—C13—C18	125.61 (11)
C14—O4—C21	116.03 (10)	O3—C13—C14	115.48 (11)
O1—C1—C6	125.38 (11)	C18—C13—C14	118.90 (11)
O1—C1—C2	115.50 (11)	O4—C14—C15	124.66 (12)
C6—C1—C2	119.12 (11)	O4—C14—C13	115.42 (11)
O2—C2—C3	124.79 (12)	C15—C14—C13	119.91 (12)
O2—C2—C1	115.21 (11)	C14—C15—C16	121.15 (12)
C3—C2—C1	119.99 (12)	C14—C15—H15	119.3 (10)
C2—C3—C4	121.06 (12)	C16—C15—H15	119.6 (10)
C2—C3—H3	119.0 (9)	C17—C16—C15	118.61 (12)
C4—C3—H3	119.9 (9)	C17—C16—C22	121.67 (12)
C5—C4—C3	118.47 (12)	C15—C16—C22	119.70 (12)
C5—C4—C10	121.09 (12)	C16—C17—C18	120.79 (12)
C3—C4—C10	120.43 (12)	C16—C17—H17	120.3 (8)
C4—C5—C6	120.97 (12)	C18—C17—H17	118.9 (9)
C4—C5—H5	120.3 (9)	C13—C18—C17	120.59 (12)
C6—C5—H5	118.7 (9)	C13—C18—H18	119.4 (9)
C1—C6—C5	120.34 (12)	C17—C18—H18	120.0 (9)
C1—C6—H6	120.2 (9)	O3—C19—C20	107.42 (9)
C5—C6—H6	119.4 (9)	O3—C19—H19A	109.0 (9)
O1—C7—C8	107.63 (9)	C20—C19—H19A	112.1 (8)
O1—C7—H7A	109.5 (8)	O3—C19—H19B	109.9 (9)
C8—C7—H7A	110.4 (9)	C20—C19—H19B	110.6 (9)
O1—C7—H7B	109.4 (9)	H19A—C19—H19B	107.9 (12)
C8—C7—H7B	111.6 (9)	C19—C20—C19^ii^	113.61 (15)
H7A—C7—H7B	108.2 (12)	C19—C20—H20B	110.4 (9)
C7—C8—C7^i^	114.11 (15)	C19^ii^—C20—H20B	107.3 (9)
C7—C8—H8B	106.1 (9)	C19—C20—H20B^ii^	107.3 (9)
C7^i^—C8—H8B	110.0 (9)	C19^ii^—C20—H20B^ii^	110.4 (9)
C7—C8—H8B^i^	110.0 (9)	H20B—C20—H20B^ii^	107.6 (18)
C7^i^—C8—H8B^i^	106.1 (9)	O4—C21—H21A	110.7 (9)
H8B—C8—H8B^i^	110.5 (18)	O4—C21—H21B	105.3 (10)
O2—C9—H9A	111.2 (10)	H21A—C21—H21B	109.0 (14)
O2—C9—H9B	104.5 (9)	O4—C21—H21C	111.0 (10)
H9A—C9—H9B	109.1 (13)	H21A—C21—H21C	111.5 (14)
O2—C9—H9C	110.3 (10)	H21B—C21—H21C	109.1 (13)
H9A—C9—H9C	111.2 (14)	C23—C22—C16	113.46 (11)
H9B—C9—H9C	110.5 (13)	C23—C22—H22A	107.1 (10)
C11—C10—C4	116.06 (12)	C16—C22—H22A	109.6 (10)
C11—C10—H10A	108.5 (9)	C23—C22—H22B	109.9 (9)
C4—C10—H10A	109.5 (10)	C16—C22—H22B	108.1 (10)
C11—C10—H10B	106.7 (10)	H22A—C22—H22B	108.7 (14)
C4—C10—H10B	108.3 (10)	C24—C23—C22	125.43 (15)
H10A—C10—H10B	107.5 (14)	C24—C23—H23	119.7 (11)
C12—C11—C10	127.94 (14)	C22—C23—H23	114.8 (11)
C12—C11—H11	118.0 (11)	C23—C24—H24A	120.3 (11)
C10—C11—H11	114.0 (11)	C23—C24—H24B	122.2 (12)
C11—C12—H12A	121.0 (11)	H24A—C24—H24B	117.5 (17)
			
C7—O1—C1—C6	−3.97 (17)	C19—O3—C13—C18	2.61 (18)
C7—O1—C1—C2	175.08 (10)	C19—O3—C13—C14	−176.67 (10)
C9—O2—C2—C3	11.70 (18)	C21—O4—C14—C15	−14.30 (18)
C9—O2—C2—C1	−167.55 (11)	C21—O4—C14—C13	164.57 (11)
O1—C1—C2—O2	−1.88 (16)	O3—C13—C14—O4	2.86 (16)
C6—C1—C2—O2	177.24 (11)	C18—C13—C14—O4	−176.47 (11)
O1—C1—C2—C3	178.83 (11)	O3—C13—C14—C15	−178.22 (11)
C6—C1—C2—C3	−2.05 (18)	C18—C13—C14—C15	2.45 (18)
O2—C2—C3—C4	−178.74 (12)	O4—C14—C15—C16	177.52 (12)
C1—C2—C3—C4	0.48 (19)	C13—C14—C15—C16	−1.29 (19)
C2—C3—C4—C5	1.21 (19)	C14—C15—C16—C17	−0.69 (19)
C2—C3—C4—C10	−179.70 (12)	C14—C15—C16—C22	−178.93 (12)
C3—C4—C5—C6	−1.35 (19)	C15—C16—C17—C18	1.52 (19)
C10—C4—C5—C6	179.57 (12)	C22—C16—C17—C18	179.71 (12)
O1—C1—C6—C5	−179.06 (11)	O3—C13—C18—C17	179.10 (12)
C2—C1—C6—C5	1.92 (19)	C14—C13—C18—C17	−1.65 (19)
C4—C5—C6—C1	−0.22 (19)	C16—C17—C18—C13	−0.34 (19)
C1—O1—C7—C8	178.57 (10)	C13—O3—C19—C20	−179.34 (10)
O1—C7—C8—C7^i^	56.52 (7)	O3—C19—C20—C19^ii^	−56.61 (7)
C5—C4—C10—C11	−113.66 (15)	C17—C16—C22—C23	126.83 (14)
C3—C4—C10—C11	67.28 (17)	C15—C16—C22—C23	−55.00 (17)
C4—C10—C11—C12	−1.4 (2)	C16—C22—C23—C24	111.83 (17)

Symmetry codes: (i) −*x*+3/2, *y*, −*z*+1/2; (ii) −*x*+1/2, *y*, −*z*+1/2; (iii) *x*, *y*−1, *z*; (iv) −*x*+2, −*y*, −*z*+1; (v) −*x*+3/2, *y*+1, −*z*+1/2; (vi) *x*, *y*+1, *z*; (vii) −*x*+1, −*y*, −*z*+1; (viii) −*x*+3/2, *y*−1, −*z*+1/2; (ix) −*x*+2, −*y*+1, −*z*+1; (x) *x*+1, *y*+1, *z*.

### Hydrogen-bond geometry (Å, º)

*Cg*1 and *Cg*2 are the centroids of benzene rings *A* (C1–C6) and *B* (C13–C18), respectively.

**Table:**

*D*—H···*A*	*D*—H	H···*A*	*D*···*A*	*D*—H···*A*
C7—H7*A*···*Cg*1^iii^	1.003 (16)	2.759 (15)	3.6170 (15)	144.1 (12)
C19—H19*B*···*Cg*2^vi^	0.992 (16)	2.739 (15)	3.5816 (15)	143.0 (11)

Symmetry codes: (iii) *x*, *y*−1, *z*; (vi) *x*, *y*+1, *z*.
